# Dental Management of a Child with Joubert Syndrome

**DOI:** 10.22037/ijcn.v16i2.28713

**Published:** 2022-03-14

**Authors:** Rezvan RAFATJOU, Sima TORKAMAN, Fahimeh DANESHYAR

**Affiliations:** 1 Department of Pediatric Dentistry, Faculty of Dentistry, Hamedan University of Medical Sciences, Hamedan, Iran.; 2 Department of Pediatric Dentistry, Faculty of Dentistry, Kermanshah University of Medical Sciences, Kermanshah, Iran.

**Keywords:** Joubert syndrome, Molar tooth sign, Hypoplasia

## Abstract

Joubert syndrome is a rare genetic autosomal recessive disorder, which is estimated to occur in 80,000 to 100,000 live births. Brain magnetic resonance imaging (MRI) indicating the molar tooth sign can be an important indicator of Joubert syndrome. Prognosis depends on the severity and extent of respiratory disorder immediately after birth. Herein, we report the case of a five-year-old boy with Joubert syndrome, who visited the hospital with his parents. He was unable to chew because of toothache and tooth decay. Considering his poor clinical condition and inability to cooperate, dental procedures were performed under anesthesia at the hospital. Generally, these patients are sensitive to the respiratory effects of anesthetics, such as opiates and nitrous oxide; therefore, they should be avoided. In the present case, sevoflurane gas was used to induce general anesthesia. Advanced dental caries have been observed in previous cases, which might be attributed to dental hypoplasia and inability to observe dental and oral hygiene. Therefore, the patient’s parents must be given the necessary instructions on the observance of orodental hygiene, and regular follow-ups are necessary for dental checkups and preventive measures.

## Introduction

Joubert syndrome is a rare genetic autosomal recessive disorder. The main symptoms of this disease include hypotonia, ataxia, intellectual disability, abnormal eye movements, and alternate breathing patterns of tachypnea, and apnea during the first months of life. This disorder was first described by Joubert in 1969. Although its prevalence has not been clearly determined, it is estimated to occur in 80,000 to 100,000 live births; however, these statistics may be underestimated (1). Clinically, it is a heterogeneous disorder with a combination of neurological symptoms, involving various body organs, mainly the retina, liver, kidneys, and skeletal system; this pleiotropism is probably an indicator of the genetic background of this syndrome (2). 

In recent decades, major attention has been paid to the genetic background of Joubert syndrome. It is claimed that over 35 genes can cause this disorder (3). It resembles other ciliopathies, especially Meckel syndrome, both clinically and genetically. On the other hand, Meckel syndrome is a life-threatening malformation, associated with encephalocele, anomalies of the posterior fossa, hepatic ductal plate malformation, and polycystic kidneys (4). Hypotonia, abnormal eye movements, and developmental delay fall into the list of differential diagnoses for Joubert syndrome. The simultaneous occurrence of these disorders with abnormal breathing patterns strengthens the clinical suspicion of this syndrome. Magnetic resonance imaging (MRI) is needed for a definite diagnosis. 

Immediately after birth, the prognosis of patients depends on the severity and extent of respiratory disorders. Commonly, long-term duration of respiratory apnea may be life-threatening and require respiratory assistance. However, most of these respiratory disorders subside during the first few months or years of life. Nutritional problems may also be seen in some of these patients. The prognosis largely depends on hepatic and renal complications, and without timely diagnosis and treatment, the patient may expire (5). 

If a child has a dental problem, general anesthesia becomes necessary for safe, efficient, and effective pediatric care. This technique is commonly advised due to the patient’s lack of emotional/psychological maturity, physical/intellectual disability, or other life-threatening medical problems that prevent the patient’s informed relaxation and cooperation.

## Case Report

**Figure 1:**
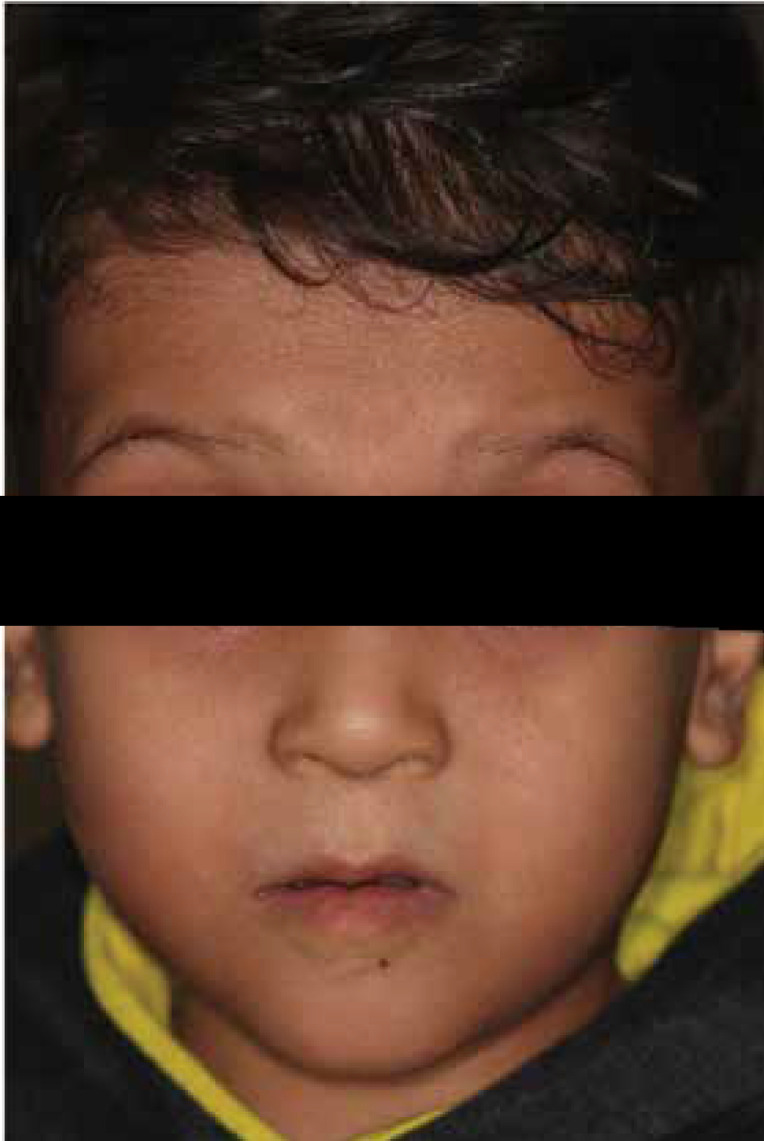
The clinical appearance of the patient (a five-year-old boy) with ptosis and high rounded eyebrows

A five-year-old boy was admitted to Hamedan School of Dentistry (Hamedan, Iran) by his parents with a chief complaint of inability to chew due to dental caries and toothache. There was no family history of any diseases, and the parents were non-consanguineously married. Also, the mother’s pregnancy and delivery had been without complications. The child’s birth weight was 2.5 kg, and his head circumference was normal. He suffered from a cognitive disorder. Upon general physical examination and history taking, the sluggish movement of limbs, developmental delay, and inability to speak were confirmed (Figure 1). 

**Figure 2:**
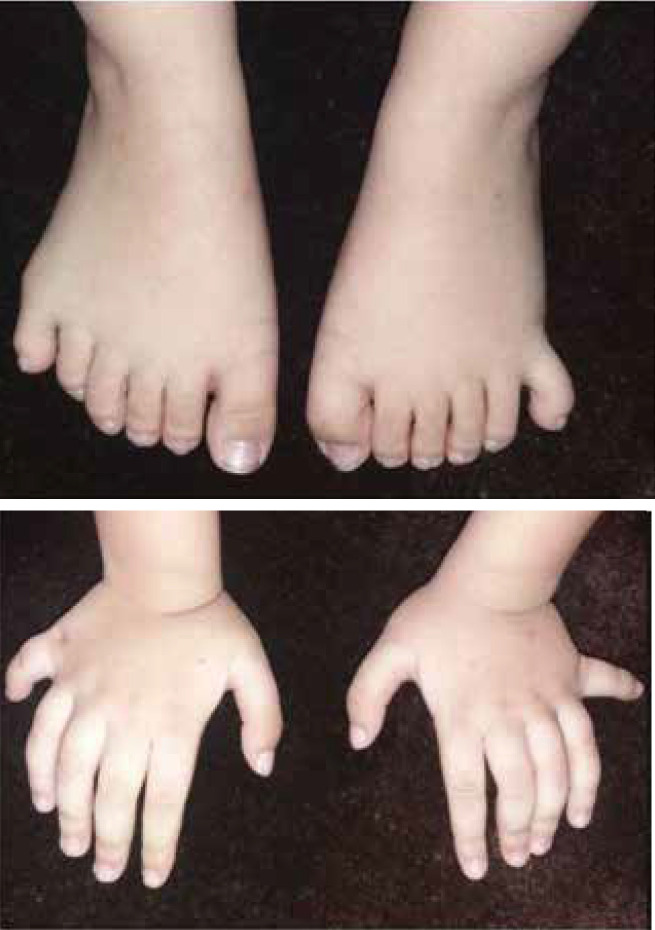
Images of polydactyly of the hands and feet

**Figure 3:**
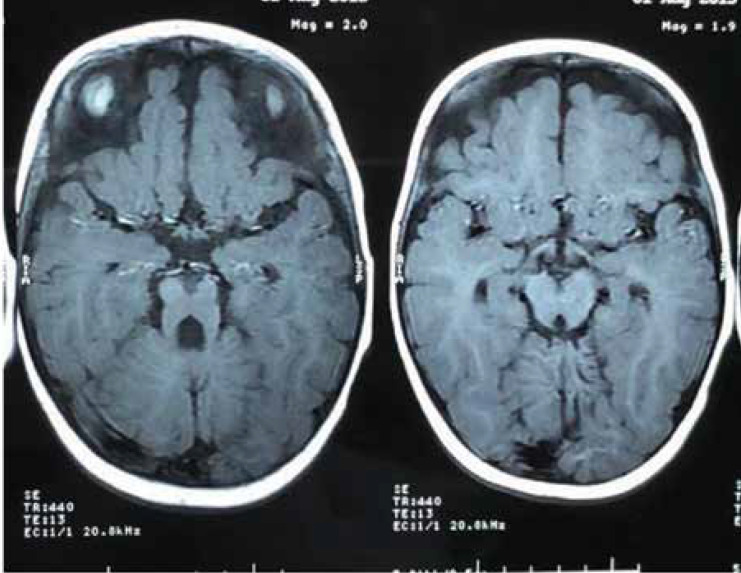
Brain magnetic resonance image (MRI) of the patient indicating the characteristic molar tooth sign

The ultrasound did not indicate any involvement of the liver, pancreas, kidneys, or spleen. The child showed polydactyly in both hands and feet, as well as mild scoliosis (Figure 2). Despite no apparent deformities in the lower limbs, he was unable to walk. The ophthalmological examination revealed his inability to focus on objects or follow mobile objects. Further examination by a neurologist indicated that he was a known case of Joubert syndrome. MRI of the brain revealed cerebellar vermis hypoplasia and a thick superior cerebellar peduncle, suggesting the molar tooth sign (Figure 3).

**Dental examination:**

The orodental examination revealed advanced caries in the molars and anterior teeth of both upper and lower jaws. The patient’s gums were inflamed, and apparently, he had poor dental hygiene. The radiographic examination of the teeth indicated that his permanent teeth had enamel hypoplasia; however, no tooth was missing. According to the patient’s poor clinical condition and inability to cooperate, dental procedures were performed under anesthesia at the hospital. Before the procedure, all necessary paraclinical examinations were prescribed. Since the patient was anemic (based on the laboratory results), the necessary consultation was made to ensure that anemia would not pose any problems to anesthesia; the results indicated no hindrance to the procedure. 

The patient was examined by the anesthetist, and his current health status and medical history were examined. Recommendations were also made on the presurgical fasting duration. Since certain anesthetic gases can trigger apnea, inhalational sevoflurane was administered for the patient. The palatopharyngeal region was sealed with a wet sterile gauze. Untreatable teeth with a dentoalveolar abscess were extracted based on the clinical and radiographic examinations before surgery. Next, pulp therapy and restoration of teeth were carried out. The anesthetist terminated anesthesia induction, and the patient was kept under observation. 

**Figure 4:**
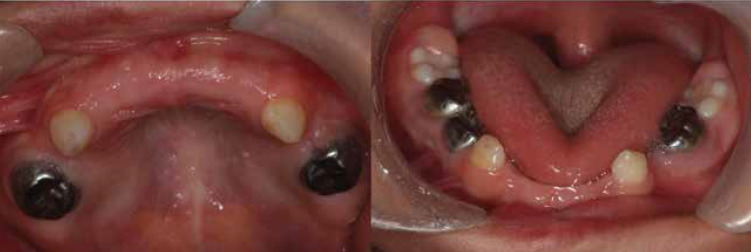
Intraoral images of the patient after dental treatment

**Figure 5:**
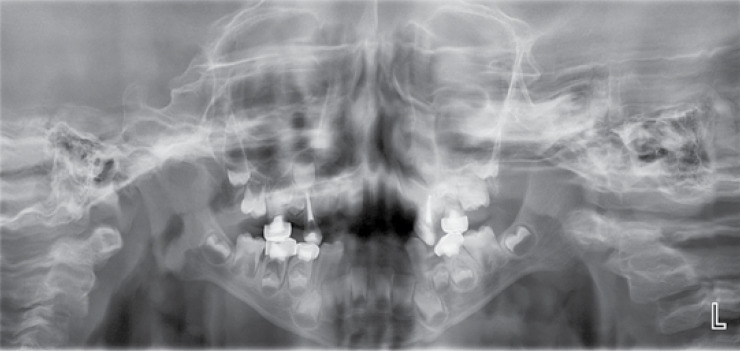
A panoramic radiographic image indicating the treated deciduous teeth and hypoplasia of permanent teeth

No complications were observed upon the induction of general anesthesia, and the child was discharged the next day in complete health with normal vital signs. Postoperative instructions were given to the parents. The necessary instructions were also given on the maintenance of dental hygiene and prevention of dental caries. Currently, the patient has no renal complications, and fluoride varnish will be applied every three months in the follow-up visits (Figures 4-5).

## Discussion

Joubert syndrome is recognized by the following characteristics: cerebellar vermis hypoplasia, developmental delay, hypotonia, and abnormal eye movements and/or breathing patterns. It is an autosomal recessive disorder that mostly occurs in males (1). The present case was also a boy, visiting the dental clinic with his parents. One of the differential diagnoses for this disorder is COACH syndrome, characterized by symptoms, such as cerebellar vermis hypoplasia, oligophrenia, coloboma, hepatic fibrosis, and congenital ataxia (6); however, these symptoms were absent in our patient. 

Hypotonia is observed in almost all cases of Joubert syndrome in the neonatal period or during infancy. Although this abnormal finding is present in many neuropediatric disorders, the combination of hypotonia, irregular breathing patterns, and altered eye movements strongly suggests a diagnosis of Joubert syndrome and encourages clinicians to perform brain MRI. The molar tooth sign is mandatory for the diagnosis of this disease. Developmental abilities, especially language and motor skills, are delayed to varying degrees in all these patients. Moderate to severe intellectual disability is common, although many patients are able to attend special schools, learn special occupational skills, and work in protected environments (7). 

Our patient showed hypotonia and abnormal eye movements; he was unable to sit without support. Based on the MRI findings, he was a known case of Joubert syndrome and was unable to talk and walk. In the literature, polydactyly has been associated with this disorder at a prevalence rate of 8% to 16%. The most common form of this syndrome is postaxial polydactyly, in which the digits of both hands and feet are involved. Mild to severe scoliosis may be one of the symptoms of this syndrome, which can be associated with certain degrees of hypotonia in the early years of childhood, whereas structural anomalies of the vertebrae are uncommon (5). Similarly, we observed polydactyly and some degree of scoliosis in our patient. 

Patients with Joubert syndrome are sensitive to the respiratory effects of anesthetics, such as opiates and nitrous oxide; therefore, they should not be administered (8). In our patient, we used sevoflurane gas to induce general anesthesia. Since the recurrence rate is 25%, prenatal screening and consultation are necessary. Renal problems are seen in 25% of these patients. Based on our investigations, the patient did not have any renal problems. Advanced dental caries have been reported in the literature, which might be attributed to dental hypoplasia and inability to observe dental and oral hygiene.

The enamel formation process is complex and may be influenced by systemic and environmental conditions, associated with enamel defects. In many studies, the association between early childhood caries and enamel defects has been demonstrated (9). Joubert syndrome may be described as a systemic disease, affecting the process of enamel formation and leading to caries development. Despite reports of dental caries in these patients, it has not been investigated in any previous studies. 

The prenatal diagnosis of high-risk pregnancies is made using serial ultrasounds, as well as fetal MRI at 20 to 22 weeks of gestation. Pediatricians and primary care physicians can help detect Joubert syndrome earlier and offer prenatal genetic consultations to patients with confirmed genetic mutations. They can also advise appropriate physiotherapeutic and rehabilitative plans for the management of this disorder, which can undoubtedly improve the natural motor and functional development of the patient (10). 

In conclusion, if a child has dental problems and is unable to cooperate, dental procedures should be conducted under general anesthesia. Therefore, the patient’s parents must be given the necessary instructions on the observance of orodental hygiene, and regular follow-ups are essential for dental checkups and preventive measures. 

## Informed Consent

A written informed consent form was obtained from the patient’s parents to publish this article and his images. We would like to thank the patient for his cooperation. 

## Author’s Contribution

All the authors contributed equally to the present study and were involved in drafting and review of the manuscript.

## Conflict of Interest

The authors declare that there is no conflict of interest.
